# Childhood Respiratory Illnesses Before and After COVID-19 Pandemic Restrictions

**DOI:** 10.7759/cureus.72957

**Published:** 2024-11-04

**Authors:** Melanie M Randall, Jennifer Raae-Nielsen, Christin J Tu, Besh R Barcega, Timothy P Young, Lance A Brown

**Affiliations:** 1 Pediatric Emergency Medicine, Loma Linda University Medical Center, Loma Linda, USA

**Keywords:** covid-19, lockdown restrictions, masking, pediatric, respiratory infections

## Abstract

Introduction

With the COVID-19 pandemic, multiple studies described a significant drop in common respiratory viruses in children with the lockdown and restrictions. With the lifting of pandemic precautions, we had the ability to observe new patterns of respiratory illnesses in children and emergency department visits.

Materials and methods

We studied all respiratory nucleic acid amplification test results in emergency patients from a large metropolitan children's hospital from the years 2018 to 2023. The test included adenovirus, coronaviruses HKU1, NL63, 229E, OC43, metapneumovirus, rhinovirus or enterovirus, influenza A and B, parainfluenza virus types 1-4, respiratory syncytial virus, *Bordetella pertussis*,* Bordetella parapertussis*, *Chlamydophila pneumoniae*, and *Mycoplasma pneumoniae*. Coronavirus SARS CoV-2 became part of the respiratory panel in November of 2020. We reviewed pediatric emergency department census data to describe the trends before, during, and after COVID-19 pandemic restrictions.

Results and conclusions

Prior to 2020, there was a median of 1080 tests performed per week with an average positivity rate of 3.7-4.1%. During 2020, this dropped to 486 tests per week with a positivity rate of 1.74%. In 2021, after schools reopened, the median number of tests was 589 per week, with a positivity rate of 4.07%. After schools reopened without masks, the median tests per week were 817, with a positivity rate of 4.71%. Emergency department census data showed a large rebound in 2021 and 2022, with significantly earlier census peaks in these years. Common pediatric respiratory illnesses had an early seasonal spike in the years after the restrictions were lifted, with the most significant being the year in which local schools stopped wearing masks.

## Introduction

With the onset of the COVID-19 pandemic in 2020, extreme efforts were made to prevent its spread throughout the world. These included school and business closures, social distancing, mandatory use of masking, and an overall decrease in human interactions and movement of population. We have previously described the significant and unusual drop in common respiratory viruses in children that coincided with pandemic restrictions in the spring of 2020 [1]. This was confirmed by other studies [2-8].

With the lifting of pandemic precautions in the years after, we had a unique opportunity to observe new patterns of common respiratory illnesses in children as schools and businesses reopened and masks were no longer mandatory. There was concern by healthcare providers regarding the immunity debt that was accumulating in the community [9]. An immunity debt is caused by prolonged non-exposure to infectious pathogens, which then leads to extremely large and unseasonal surges of respiratory illnesses [9-11]. Some studies showed significant surges of RSV, influenza, and bronchiolitis ICU admissions after the easing of COVID-19 restrictions [2,6,12-14]. With this study, we aimed to describe the trends of all pediatric respiratory illnesses before and after the COVID-19 restrictions and the corresponding emergency department (ED) census.

## Materials and methods

We performed a retrospective study of all nasopharyngeal respiratory panel results from patients less than 18 years of age in the pediatric ED at Loma Linda University Children’s Hospital from 2018 to 2023. Our children’s hospital is a tertiary receiving center, serving a population of approximately 4.5 million people from two counties. The pediatric ED has approximately 45,000 visits per year.

We collected data for each of the five years, starting from August 31 to May 30, representing each year’s approximate respiratory season. The respiratory panel used is a nucleic acid amplification test. It includes the following respiratory pathogens: adenovirus, coronaviruses HKU1, NL63, 229E, OC43, metapneumovirus, rhinovirus or enterovirus, influenza A and B, parainfluenza virus types 1-4, respiratory syncytial virus, *Bordetella pertussis*, *Bordetella parapertussis*, *Chlamydophila pneumoniae*, and *Mycoplasma pneumoniae*. Coronavirus SARS CoV-2 became part of the respiratory panel on November 11, 2020. We reported each individual test from the panel that was positive during the study period, thus one patient could have multiple positive tests if they had co-infections. In our hospital, the panel is performed on all children admitted to the hospital for respiratory complaints. For patients who can be discharged home, the panel is ordered at the discretion of the ED provider.

We collected census data including daily patient encounters in the pediatric ED during the study period. This project was approved by the Loma Linda University Medical Center institutional review board.

## Results

**Figure 1:**
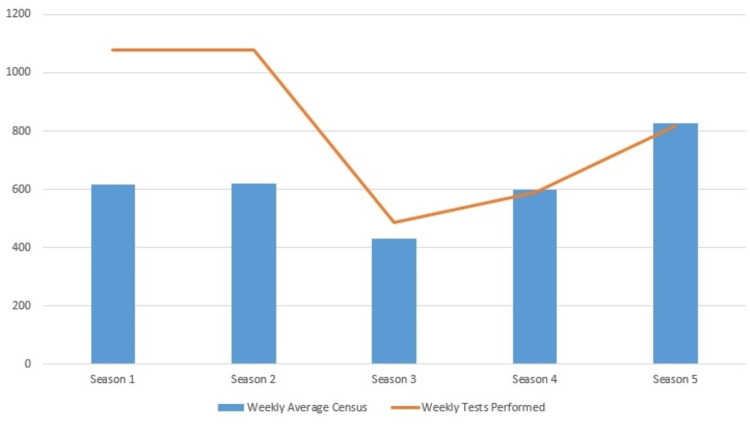
Average weekly tests performed X-axis: season, Y-axis: number of weekly tests Season 1: 2018-2019, Season 2: 2019-2020, Season 3: 2020-2021, Season 4: 2021-2022, Season 5: 2022-2023

**Figure 2:**
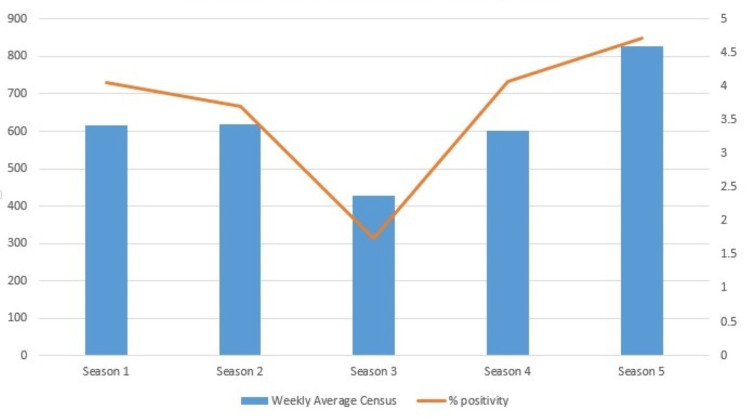
Average weekly percent positivity X-axis: season, Y-axis left: weekly average census, Y-axis right: percent positivity Season 1: 2018-2019, Season 2: 2019-2020, Season 3: 2020-2021, Season 4: 2021-2022, Season 5: 2022-2023

In season 1 (2018-2019), a median of 1,080 individual respiratory pathogen tests were conducted weekly (IQR 799-1428), with a median positivity rate of 4.06% (IQR 3.50-4.77%). During season 2 (2019-2020), the weekly median remained at 1,080 tests (IQR 558-1980) with a median positivity rate of 3.70% (IQR 3.14-5.05%). In season 3 (2020-2021), there was a decrease to a median of 486 tests per week (IQR 414-552) and a median positivity rate of 1.74% (IQR 1.44-2.47%). Season 4 (2021-2022) saw a weekly median of 589 tests (IQR 133-1083) with a median positivity rate of 4.07% (IQR 3.33-4.63%). By season 5 (2022-2023), the median number of weekly tests was 817 (IQR 684-1,197), with a median positivity rate of 4.71% (IQR 4.01-5%). These numbers are shown in Figures 1 and 2.

**Figure 3:**
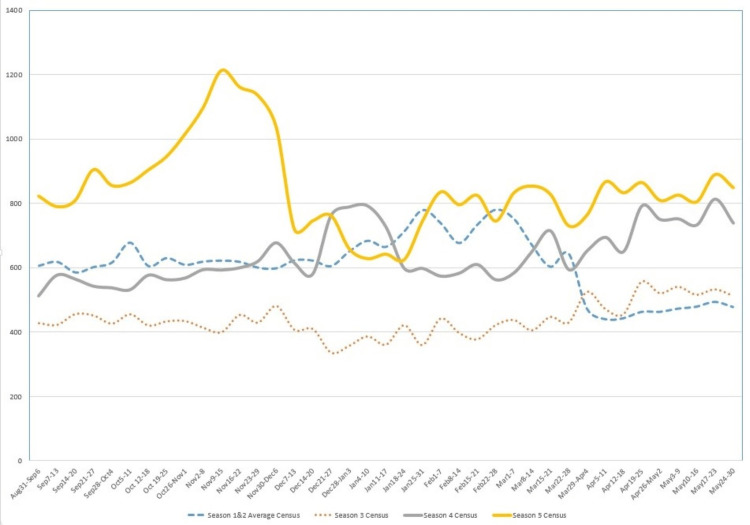
ED Census by Week X-axis: week, Y-axis: weekly census Season 1: 2018-2019, Season 2: 2019-2020, Season 3: 2020-2021, Season 4: 2021-2022, Season 5: 2022-2023

The average weekly ED census in seasons 1 and 2 was 617 and 618, respectively. During season 3, the average weekly census was 429. During season 4, the average weekly census was 600. During season 5, the average weekly census was 828 (Figure 3). Season 5 had a large and earlier census peak that occurred in November, compared to other seasons (Figure 3).

## Discussion

Our study found a quick rebound in the number of common respiratory illnesses in the years after the pandemic restrictions. After restrictions were lifted, the ED census showed significant increases from our previous baseline. This was evident first in the winter of 2021, a few months after schools re-opened for the first time. The largest increase was in the fall of 2022, a few months after schools opened without masks. Daily census records were constantly being broken at our location. Pediatric healthcare systems across the county were quickly inundated and overwhelmed during this time [15,16].

With the COVID-19 lockdown, the percent positivity of respiratory testing decreased significantly. During the following season, it quickly rebounded close to pre-lockdown numbers. During the 2022-2023 season, it often exceeded the previous baseline. We found a decrease in the number of respiratory panels ordered after the pandemic, even when the census increased. This was most likely due to a change in our practice, given the shortage of many items, including respiratory testing.

Our study points to the association between population-based changes and large swings in health care utilization. After one and a half years without in-person education, suddenly, all schools were open. This coincided with the ED census quickly rebounding with a sharp spike in December 2021. The following year, when masks were removed in schools, there was an enormous early surge in ED pediatric volume in October and November 2022. Compared to the more gradual census increases prior to COVID-19, the years following had surges that were earlier and steeper.

This data shows that distancing and use of masks initially is associated with a severe and measurable decrease in all common respiratory pathogens and ED census. However, the low volumes during this period may be offset by a higher increase later due to the immunity debt [9]. During future pandemics, if we decide to mask children, the cost will likely happen when the masks come off. We must be ready ahead of time to commit the needed resources for the inevitable impact on the healthcare system.

Limitations to this study include the fact that it is a retrospective cross-sectional study. We have made conclusions based on the ED census and respiratory pathogen testing without more granular information on individual patients and their disease processes and outcomes. There is practice variability at our site with respiratory testing, and this has the potential to contribute to changes in a number of patients tested. We also are unable to control for changes in patient and parental actions, such as seeking out emergency care, given the evolving information during and after the COVID-19 pandemic.

## Conclusions

Common pediatric respiratory illnesses and ED census had an early seasonal spike in the years after COVID-19 restrictions were lifted. We found a significant rebound in the ED census and an early spike in respiratory illnesses the year that schools reopened. The most significant increase was the year in which local schools stopped wearing masks. In this season, we had record-breaking ED census numbers very early in November. 

Healthcare providers must be aware that in any future pandemics, the use of masks and social distancing may lead to an immunity debt. In the short term, we may be able to limit the spread of a pandemic illness, but in the long term, we may delay huge surges in patients and respiratory illnesses. This is especially true in the pediatric population, for which we will need to expect significant census escalations and strains to the healthcare system when restrictions end.
